# The Potential of Curcumin-Capped Nanoparticle Synthesis in Cancer Therapy: A Green Synthesis Approach

**DOI:** 10.3390/nano12183201

**Published:** 2022-09-15

**Authors:** Jeaneen Venkatas, Aliscia Daniels, Moganavelli Singh

**Affiliations:** Nano-Gene and Drug Delivery Group, Discipline of Biochemistry, University of KwaZulu-Natal, Private Bag X54001, Durban 4000, South Africa

**Keywords:** curcumin, green chemistry, cancer, nanoparticles, delivery systems

## Abstract

Cancer nanotherapeutics is an important field of research which utilizes nanomaterials as an approach to cancer therapy. Nano-mediated therapeutic delivery systems overcome the adverse side effects of traditional cancer treatment methods. Nanoparticles (NPs) are considered excellent tumor-targeting vehicles due to their compact and variable size, large surface area, ability to load several genes and drugs, and mediation of increased therapeutic payload uptake. Despite the rapid development of nanotechnology, there is growing concern regarding the possible long-term side effects of NPs on the environment and human health. Green chemistry using plant materials, such as curcumin, is a sustainable alternative to conventional reduction methods and confers dual reducing and capping properties. Curcumin is a bioactive compound isolated from the rhizome of the *Curcuma longa* plant, which exhibits various medicinal properties. Curcumin-capped NPs exhibit increased solubility, bioavailability, therapeutic indices, and antitumor properties. This review highlights the potential and antitumor properties of economical, simple, and eco-friendly curcumin-synthesized and capped NPs for the localized delivery of therapeutic genes and drugs to the cancer tumor microenvironment with fewer adverse side effects.

## 1. Introduction

Cancer remains a leading cause of death worldwide with a poor prognosis. It is one of the deadliest diseases in human history with an inferior prognosis [1]. Cancer transforms healthy cells through inherited and acquired genetic mutations conferring growth and survival advantages and ultimately generates malignant neoplasms that invade adjacent tissues and spread to distant organs [2]. In 2020, 19.3 million novel cases and 10.0 million deaths were reported as a result of cancer, according to the World Health Organization (WHO), making the disease the second most common cause of death worldwide. The WHO further predicted that the number of cancer patients will increase to 29.4 million annually by 2040 [3]. The high global incidence and mortality rate of this non-communicable disease costs the economy billions of dollars [4]. Cancer displays great complexity at the cellular, epigenetic, and genetic levels, resulting in a reduction in long-term efficiency and the development of multiple drug resistances, non-specificity, dosage limitations, and adverse side effects. Conventional cancer treatment is also limited by the tumors’ pathological characteristics and the abnormal architecture of blood vessels [5].

Nanotherapeutics can enhance therapeutic indices, targeting, biodistribution, oral bioavailability, and aqueous solubility, making them promising candidates to challenge conventional cancer treatments [6,7]. Nanoparticles (NPs) have many uses in nutraceuticals, pollution management, and gene or drug delivery systems [8]. Various chemical, physical, and biological methods have been used to synthesize NPs. Although physical and chemical means of NP synthesis yield well-defined NPs, hazardous and expensive reducing reagents are frequently utilized, restricting upscaling and possibly damaging the environment [9,10].

Biological or green synthesis of NPs has gained much attention, as it employs plant extracts to synthesize NPs in an ecologically sound, environmentally friendly, and non-toxic manner [11]. In addition, using biological resources for NP manufacturing does not require complicated processes. Globally, a host of botanical moieties have been successfully used to synthesize different NPs [12]. Many of these NPs contain biologically active compounds that display medicinal properties [13], with curcumin being one such compound.

Curcumin is the principal curcuminoid obtained from the rhizomes of *Curcuma longa*. This bioactive component has many biological and pharmacological properties, including antioxidant, hypotensive, anti-inflammatory, anticoagulant, antifertility, antiulcer, antimicrobial, antivenom, antifibrotic, antimutagenic, antidiabetic, anticarcinogenic, and most importantly anticancer activities [14]. Although the health benefits of curcumin have been well established, the full potential of curcumin in biomedical applications has yet to be fully exploited due to its crystalline structure, low solubility in water, and bioavailability (~1%) [15]. Reducing the size of the curcumin improves its solubility and, consequently, its bioavailability, with current studies aiming to increase the bioavailability of curcumin [16] specifically. This has led to the discovery of hybrid NPs, which comprise organic or inorganic NPs enveloped by curcumin [17].

Multidrug-loaded nanocarriers or curcumin NPs are a potential strategy for fighting cancer. Incorporating co-delivery systems as a feasible treatment method has projected synergistic benefits and limited undesirable effects [18]. Curcumin-capped NPs can introduce a synergistic effect by reducing the amount of the main therapeutic component needed. This can improve the therapeutic activity while reducing toxicity. Compared with NPs only and free curcumin, curcumin-capped NPs have shown a high level of cytotoxicity in some malignant cells [13]. Curcumin-containing nanocomplexes’ strong anticancer characteristics and their positive in vitro and in vivo results have prompted their in vivo investigation [19]. This review discusses curcumin’s antitumor properties and the potential of curcumin as a reducing agent in the synthesis of NPs for cancer therapy. Although anticancer studies have been conducted using curcumin, its use as a reducing or capping agent for synthesizing organic and inorganic NPs (primarily metal NPs) to produce synergistic NPs has yet to be fully explored in cancer therapy and is the main focus of this review.

## 2. Cancer Epidemiology

**Figure 1:**
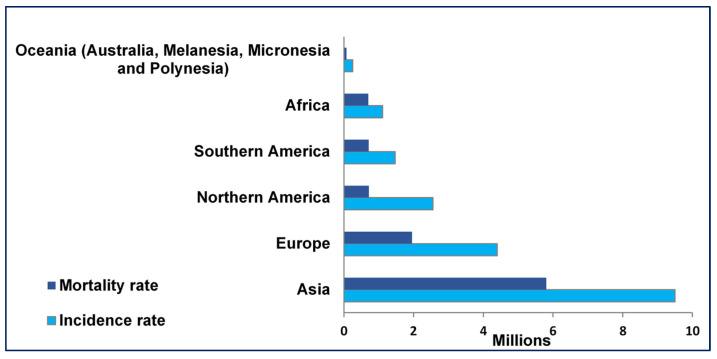
Mortality and incidence rates of cancer cases at ages <70 years in 2020. Adapted from [20].

**Figure 2:**
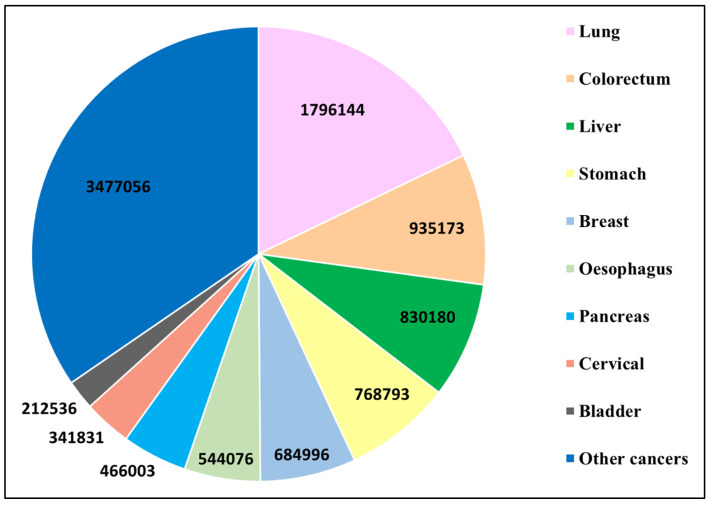
Global mortality numbers for different cancers in 2020. Adapted from [20].

Cancer is a significant cause of death worldwide, with a global incidence and mortality rate of 19.3 million and 9.6 million per year, respectively (Figure 1). Lung, colorectum, liver, stomach, breast, cervical, and bladder cancer account for more than half of the annual deaths (Figure 2) [20]. By 2017, the global cancer motility rate had almost doubled that seen in 1990 [21]. Cancer is a multi-factorial disease induced by multistage carcinogenesis involving genetic, cellular, and epigenetic abnormalities that transform healthy cells into malignant ones [6]. The evolution of cells to their cancerous state is induced by mutational damage to cancer susceptibility genes such as the tumor suppressor, DNA repair, and proto-oncogenes [22].

Oncogene mutations occur in the alleles of genes responsible for cellular growth, development, and maintenance. Mutations in the Ras family mediate cellular communication pathways, growth, and death. The *HER2* oncogenes, which mediate the growth and spread of cancer, lead to breast, ovarian, and cervical cancer [23]. Tumor suppressor genes regulate cell growth by slowing cell division, repairing DNA irregularities, and inducing apoptosis or programmed cell death. More than 50% of cancers are caused by mutations within the *p53* tumor suppressor gene [24], while germline mutations induce breast, ovarian, prostate, and pancreatic cancer in the *BRCA1* or *BRCA2* genes [25]. DNA repair genes that correct genome errors act as tumor suppressor genes. These mutations may be inherited or acquired [26].

**Figure 3:**
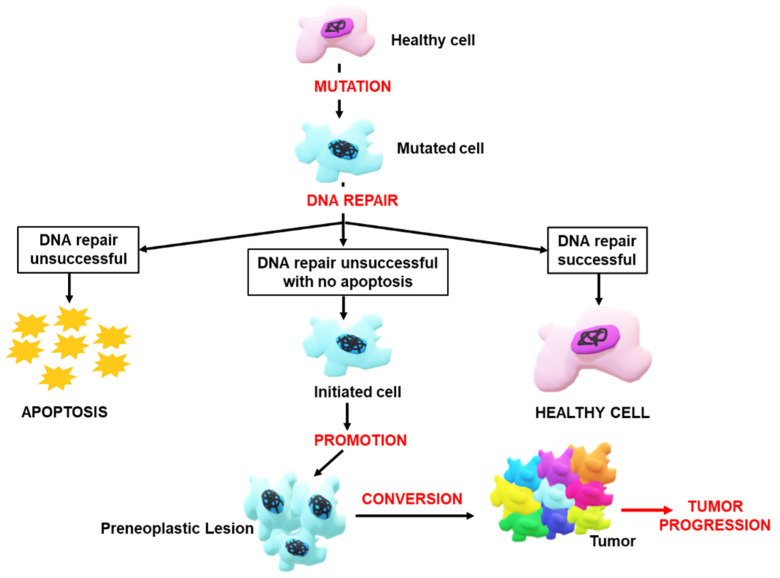
Illustration of the stages in the progression of cancer.

The first step in cancer development involves initiating or altering the cancer-susceptible genes. Once a cell is transformed, it becomes susceptible to the effects of promoters, which bind to receptors on the cell’s surface. This inhibits the apoptotic pathways, leading to uncontrollable cell proliferation [27]. As cancer progresses, the cancerous cells are transported throughout the body via the lymphatic system or bloodstream. Once these cells reach their destination, they proliferate and develop into new tumors through metastasis [28]. Figure 3 illustrates the progression of a cancer cell.

Cancer cells metastasize throughout the body via the lymphatic or blood system, where they produce tumors [8] and induce the formation of new blood vessels through the secretion of hormones. The sprouting of the new capillaries invades the surrounding tissues via angiogenesis to allow for a continuous blood supply to the tumor microenvironment [29]. The age-standardized incidence and death rates were 1.5 times higher in males than females, with individuals over 50 and residing in developing countries displaying a higher cancer incidence [21]. The increase in cancer morbidity and mortality is attributed to the population’s growth, distribution, and dominant age as well as the distribution of cancer risk factors often associated with socioeconomic development [5]. Several risk factors influence an individual’s susceptibility to cancer, including lifestyle, age, genetics, physical inactivity, weak immune systems, infections, environment, sex, dietary choices, carcinogens, and physical agents [30]. Oral cancers are induced by alcohol (7–19%), smoking or chewing tobacco (25%), and micronutrient deficiency (10–15%). In South Asian countries such as Sri Lanka, Vietnam, Indonesia, India, and Malaysia, the chewing of betel nut accounts for 50–70% of oral cancers [31]. Higher skin cancer and melanoma fatalities were observed in regions with high levels of UV exposure due to damage to the ozone layer [32].

## 3. The Promise of Cancer Nanotherapeutics

### 3.1. The Limitations of Conventional Cancer Therapy

Despite growing knowledge of cancer epigenetics, genetics, biology, and etiology, cancer treatment strategies remain suboptimal, primarily due to their off-target side effects, multiple drug resistances, and physiological barriers, limiting their optimal dosages and efficiency [5]. Furthermore, the pathological properties of tumors and their aberrant blood vessel architecture decrease traditional cancer therapy’s effectiveness [8]. Chemotherapy, the preferred choice, fails to distinguish tumor cells from non-metastatic, healthy cells and targets all rapidly dividing cells, including cells of the lymph, bone marrow, gastrointestinal tract, and hair follicles [33]. This often leads to nausea, fatigue, loss of appetite, hair loss, nephrotoxicity or kidney damage, neuropathy or peripheral neuropathy, anemia, neutropenia, and thrombocytopenia [34]. Surgical removal of tumors is often challenging, leading to further complications, such as metastasis and recurrence. The organ may also be inoperable due to the size and distribution of the tumor [35]. Hence, the intervention by nanomedicine may be beneficial in overcoming many of these challenges.

### 3.2. Overcoming Cancer Therapy Limitations with Nanomedicine

**Figure 4:**
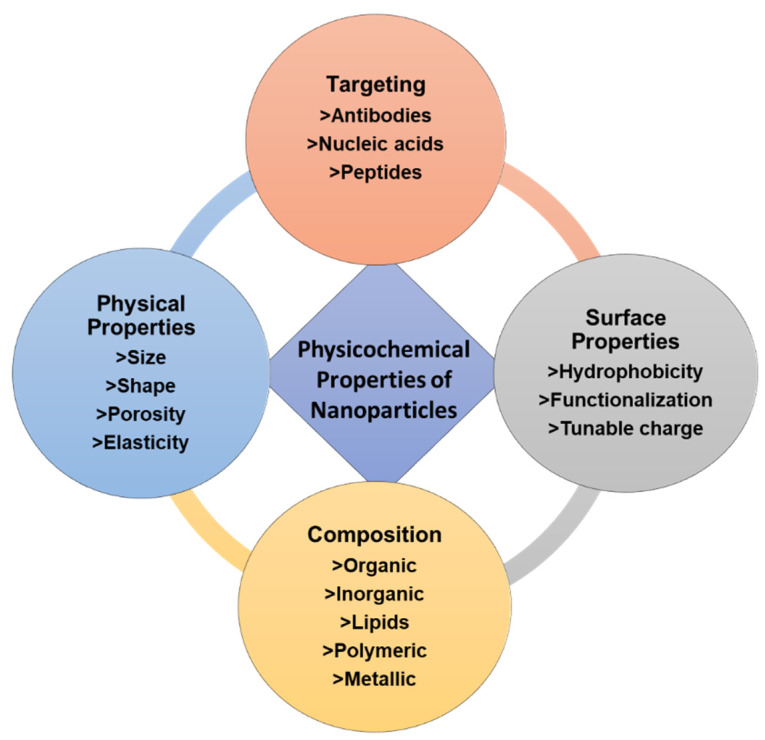
Summary of the tunable physicochemical properties of nanoparticles.

Nanomedicine is a rapidly evolving field of biotechnology. The core concept of nanotechnology dates to 1959, when Feynman demonstrated the ability of NPs to aid in the detection and pharmaceutical treatment of various human ailments [36]. The scientific understanding of the principles governing the interaction of matter with biological systems at the nanoscopic scale has advanced dramatically during the last three decades [37]. Nanomedicine utilizes nanomaterials (1–100 nm) for various biomedical applications, including tissue engineering, therapy, imaging, and diagnostics [31]. The therapeutic effect of nanomaterials is strongly influenced by their surface properties (hydrophilic-to-hydrophobic ratio and charge), physical characteristics (shape and size), and functionalization [38]. Figure 4 provides a general illustration of the favorable physicochemical properties of NPs. Carbon nanostructures, inorganic NPs (e.g., gold, silver, and selenium), lipids (e.g., liposomes), dendrimers, mesoporous silica, magnetic NPs, and polymeric NPs are among the NPs used in nanomedicine [4], with inorganic metal NPs yet to be fully explored [39].

NPs protect the therapeutic agent from opsonization and premature phagocytosis. They are absorbed at an enhanced rate by epithelial diffusion [40]. They can alter the therapeutic compound’s distribution profile and pharmacokinetics within the tumor microenvironment (TME), promoting intracellular efflux in the cells [41]. Furthermore, NPs can act as passive or active targeting agents to deliver therapeutic agents to the TME and elevate intracellular anticancer activity. Passive targeting enhances the anticancer agents’ permeability and retention effects due to the NPs’ favorable size and shape [42]. Active targeting involves the conjugation of ligands (e.g., folate) on the NP surface that can bind to receptors overexpressed on the surface of cancer cells [43,44]. This is easily facilitated by the flexible surface chemistry of the NP, which allows for the conjugation of various targeting ligands.

Multiple drug resistance (MDR) occurs when cancer cells build resistance to numerous chemotherapeutic agents, leading to drug inactivation and efflux from the malignant cell. MDR poses a severe hurdle to treatment [45]. The compact size of the NPs enables the therapeutic to be administered at safe doses, increasing their antitumor effect and overcoming tumor drug resistance [46]. This further improves the therapeutic indices and pharmacokinetics of the delivered biomolecule.

**Figure 5:**
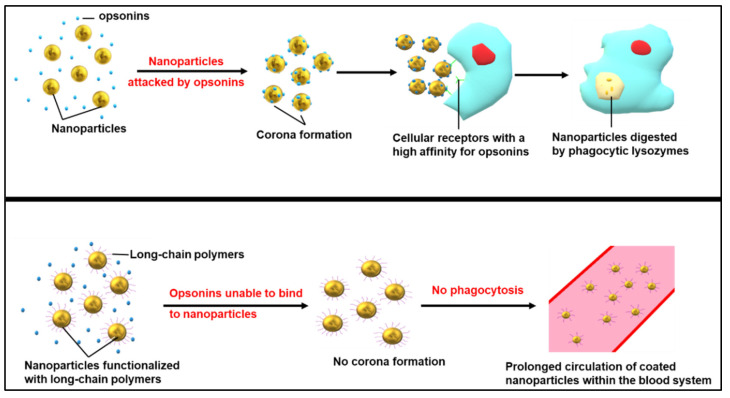
Prevention of nanoparticle opsonization using long-chained polymers such as polyethylene glycol.

NPs are amenable to steric stabilization using polyethylene glycol (PEG), which affords further stability to the NP by reducing surface-surface interactions and aggregation [47]. The PEG coating prevents the opsonization of the nanocomplexes. It allows the nanocomplex to avoid phagocytosis and clearance by the reticuloendothelial system (RES) and mononuclear phagocyte system, which are effective defense systems in the body that remove foreign material from the blood [48,49,50]. This increases the circulation time of the nanocomplex and allows the therapeutic to accumulate within the TME [51]. Figure 5 provides a simple illustration of the above process.

## 4. Green Synthesis of NPs

Various chemical, physical, and biological methods have been used to synthesize NPs, all with some advantages and disadvantages (Table 1). Although physical and chemical means of NP synthesis yield well-defined NPs, hazardous and expensive reducing reagents are frequently utilized, restricting upscaling and possibly damaging the environment [9,10]. This has created a niche for developing novel methods of NP synthesis which produce NPs with desired shapes, high thermal stability, and which use few or no toxic compounds [52,53]. This has prompted the start of the green nanotechnology revolution, where eco-friendly bioactive agents are employed in NP synthesis.

Peralta-Videa et al. (2016) stated that although plant-based green synthesis is comparable to conventional physical and chemical methods, they do not provide sufficient information on the ion’s reduction mechanisms, yield, or the stability of the NPs [54]. The mass production of NPs requires highly reactive substances and energy-consuming procedures, which are not considered environmentally friendly. The growing need to overcome these problems has prompted researchers to experiment with more economical and environmentally friendly methods. The green synthesis approach to creating NPs has gained tremendous strides as an alternative to physical and chemical syntheses [55].

**Figure 6:**
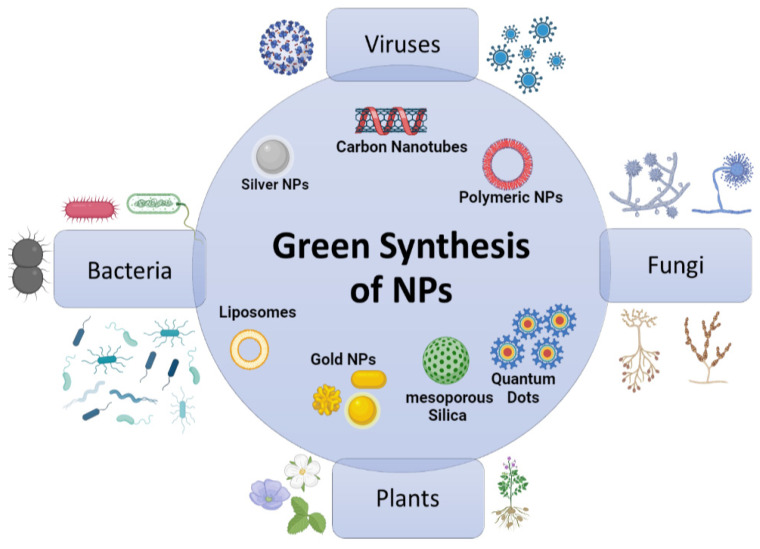
The various bioactive agents used in the green synthesis of nanoparticles.

Green synthesis can be defined as the use of various biological or bioactive agents such as plant extracts, microorganisms, fungi, and even biowastes to effectively synthesize metallic NPs in an eco-friendly and bio-reductive manner (Figure 6) [56,57]. Green synthesis vaunts a mechanism of reducing NPs while utilizing relatively low energy and maintaining a cost-effective method [58,59]. This approach, which was initially brought about due to the urgent need for “sustainable development”, requires three fundamental aspects for its success: a non-toxic reducing agent, a solvent of an environmentally friendly nature (for example, ethanol, water, and their combinations), and finally a stabilizer [60,61]. Furthermore, green synthesis offers a biocompatible, environmentally friendly, and non-toxic approach, as the byproducts and capping agents are natural to the environment, which is highly beneficial in the medical sector [56]. These NPs were reported to have a larger surface area with reduced aggregation than those produced with toxic chemical-reducing agents such as formaldehyde, hydrazine, sodium borohydride, aniline, polyvinylpyrrolidone, and sodium dodecyl sulfate [62,63,64]. In addition, these NPs showed improved photocatalytic and antioxidant capacities [54].

From the different biological agents used for green NP synthesis, microorganisms have shown some promising results. Since many inorganic metal salt ions can be toxic to microorganisms, they manufacture extra or intracellular enzymes that convert hazardous ions into harmless NPs [65]. A change in the redox state within the cell caused by foreign ions enables microbial reduced NPs to be applied in bioremediation [66]. Microorganisms can produce NPs within their cell walls. For example, the *Klebsiella aerogenes* bacterium was used to synthesize cadmium NPs [67], and *Escherichia coli* and *Deinococcus radiodurans* synthesized gold NPs [68,69]. Fungi are also attractive for large-scale production since they have a high tolerance for toxic elements and produce vast amounts of extracellular enzymes, as evidenced by silver and gold NPs extracted from *Aspergillus oryzae* and *Verticullium* [70,71]. However, compared with microbe-based synthesis, green synthesis of metallic NPs using plant-based extracts is a relatively more straightforward process which eliminates the constant maintenance of cultures [72].

NPs derived from plant-based syntheses have been employed to eradicate biofilms from clinically relevant surfaces, for targeted drug delivery, as 3D culture models, and in cancer therapy. In addition, the raw resources used in this green synthesis are renewable [73]. Plant extracts have been noted to produce NPs of varied sizes and shapes that range from rod-like to spherical, cubic, and triangular [74]. This is due to the phytochemical make-up of the plant utilized, which promotes natural stability in NP creation while doubling as a reducing agent [75]. Plant-based green synthesis is an extensive process involving the use of the whole plant biomass or extracts from different parts of the plant (stems, leaves, flowers, roots, seeds, bark, etc.) to synthesize different types of NPs [54]. The use of whole plants to synthesize metallic NPs is an intrinsic process with NPs deposited within the plant tissues, while plant extract-mediated NP synthesis occurs extracellularly. The composition of the plants affects their bioreductive ability, which in turn determines the morphology, composition, and dimensions of the nanoparticles formed [56]. Complementary to the effects of the phytochemicals present, biomolecules (carbohydrates, co-enzymes, and proteins) found in the plant extract also portray an exemplary reduction of metal salts into the desired metallic NPs. Functional compounds obtained from plant extracts, such as carboxylic acids, alkene, alkane, amine, and methylene, serve as promising reducing agents for synthesizing metallic NPs [72]. The biological activity of the synthesized NPs is primarily determined and fine-tuned by the biomaterial utilized for the stability and reduction of the metal ions [76]. The secondary metabolites of plants, such as tannins, flavones, and polyphenols, possess antioxidant, antimicrobial, and anticarcinogenic properties [77].

Apiin, isolated from *Lawsonia inermis*, reduced silver and gold metal salts via electrostatic interaction between the extract’s carbonyl groups and metal ions [78]. Phyllanthin extracted from *Phyllanthus amarus* was used to synthesize silver and gold NPs by exchanging the metal ions and the plant extract’s methoxide group [79]. Researchers discovered that compounds isolated from propolis extract could produce a broader range of NP sizes. These findings suggest that many chemicals with various reducing properties can complicate the synthesis process, impacting its simplicity and NP size distribution [80]. Spherical copper, gold, silver, selenium, and platinum of <100 nm in size were produced by extracts from either leaves, flowers, stems, fruit, or seeds from various plants [81,82,83,84,85,86,87,88,89]. However, triangular and hexagonal shapes were also reported for green synthesized gold, silver, and silver-selenium bimetallic NPs [86,88,90], while titanium dioxide NPs produced unusual tetragonal shapes [91,92].

However, there are still barriers to be overcome in relation to more conventional methods for synthesizing NPs [9]. The ability to control the shape and size of biologically synthesized NPs has been a significant impediment, since the size and shape of the NP are predetermined by the different phytochemical compositions of the plant extract [81]. Similar plants grown in other geographical areas or harvested at different times can produce varied bioactive constituents. As a result, the shape and size of the NPs produced are altered [84]. This in turn can cause a decline in their market value, as the morphology of commercially manufactured NPs is uniform. Moreover, plant extracts contain many active phytochemicals which require isolation and purification [73]. However, the benefits of green synthesis outweigh the disadvantages, providing a niche for scientists to improve their means of plant-mediated synthesis.

## 5. The Properties of Curcumin

*Curcuma longa*, also known as turmeric, is an ancient perennial plant native to India that belongs to the Zingiberaceae family. Curcuma has evolved via continuous crossbreeding and selection. Over 100 Curcuma species have been identified to date [93]. The perennial herb grows in subtropical and tropical regions worldwide and is widely cultivated in Asian countries, such as India, Taiwan, Japan, Vietnam, Indonesia, Thailand, Burma, Bangladesh, and China [94]. The *C. longa* rhizome has an oblong pyriform shape with short branches. Turmeric is considered a significant plant in Ayurvedic history, having been used to treat a broad range of ailments in Indian Ayurvedic medicine since 1900 BC, including wounds, aches and pains, sprains, gastrointestinal system disorders, and liver disorders [95]. The biological constituent has been extensively studied for its bioactivity [96]. Curcumin exhibits antimicrobial, antioxidant, anticancer, anti-inflammatory, hyperlipidemic, hepatoprotective, and neuroprotective activities [95].

**Figure 7:**
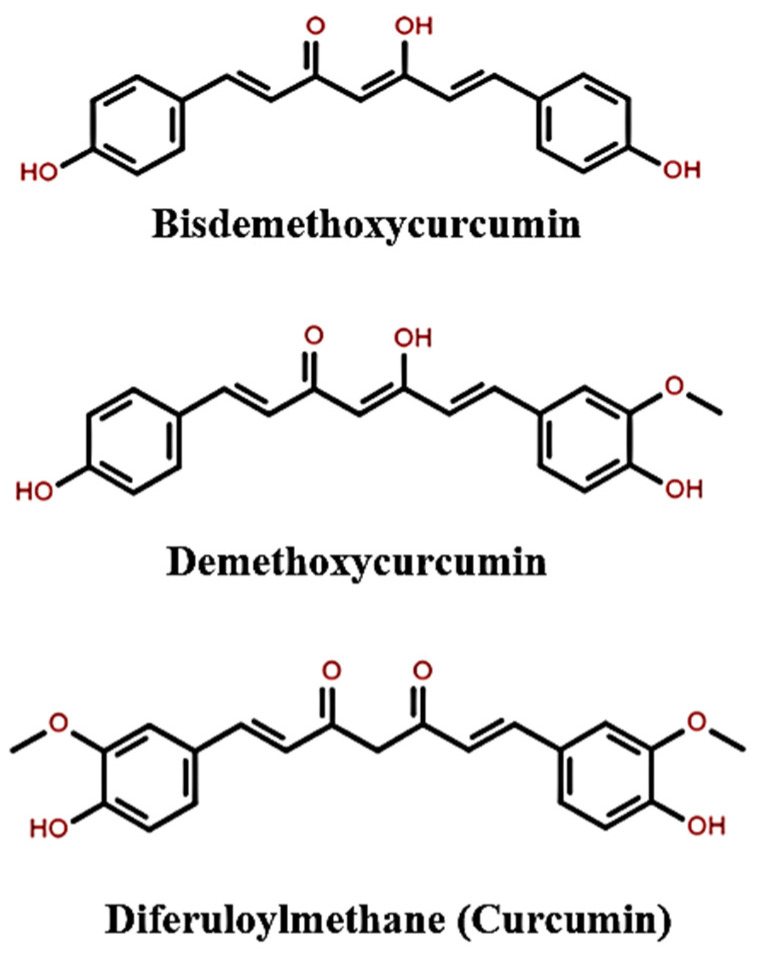
Chemical structures of bisdemethoxycurcumin, demethoxycurcumin, and diferuloylmethane (curcumin). Drawn using ChemSpider and available online: http://www.chemspider.com/Chemical-Structure.1906.html (accessed on 30 May 2021).

Turmeric can be broken down into three curcuminoids (Figure 7): bisdemethoxycurcumin, demethoxycurcumin, and diferuloylmethane [96]. The latter, a polyphenol, is a primary constituent of turmeric which accounts for turmeric’s vibrant yellow color and is commonly referred to as curcumin. The additional components of turmeric include proteins, sugars, resins, and volatile oils (zingiberone, atlantone, and turmerone) [94]. Curcumin (C_21_H_20_O_6_) comprises two polyphenolic rings connected by a C7-linker containing an unsaturated β-diketone motif [97]. The bioactive component is insoluble in water under neutral and acidic pH levels. Still, it is soluble in acetone, dimethyl sulfoxide (DSMO), and ethanol, with a melting temperature and molecular weight of 183 °C and 368.37 g∙mol^−1^, respectively [95].

Curcumin contains two tautomeric forms: keto and enol, with the former being energetically stable in both the solid and liquid phases. However, the bioactive components take the form of a bis-keto (1,7-bis(4-hydroxy-3-methoxyphenyl)-1,6-heptadiene-3,5-dione) under acidic and neutral conditions [96]. It was reported that curcumin had enhanced antioxidant properties compared with its curcuminoid counterparts. This was attributed to the transition metal chelation attaching the o-methoxy and diketone phenols [93]. Curcumin blocks the NFkB and hemeoxygenase-1 pathways, which are responsible for the structural moieties of the α, β-unsaturated diketone that serves as an acceptor in the Michael reaction. Various studies have elaborated on curcumin’s biological and pharmaceutical properties [94,98,99,100].

### 5.1. Biological and Pharmaceutical Properties of Curcumin

Curcumin is one of turmeric’s most active therapeutic agents due to its biological and pharmaceutical properties [95]. It can directly or indirectly bind to and deactivate various metals, proteins, receptors, growth factors, enzymes, and transcription factors. Direct targets include cell survival proteins, carrier proteins, protein kinases and reductases, metal ions, proteasomes, inflammatory molecules, and DNA methyltransferase 1. In contrast, indirect targets encompass proteins for cell survival, transcription factors, mediators of inflammation, enzymes, receptors growth factors, adhesion molecules, and cell cycle proteins [92].

The antimicrobial property of curcumin is renowned and influenced by its interaction with the FtsZ protein. The FtsZ protein is responsible for an essential stage of cell division in most prokaryotic species [94,101]. Reports conclude that curcumin’s hydroxyl and methoxy groups are directly linked to antimicrobial activity. The oxygen molecules of these functional groups linked to curcumin’s phenolic rings catalyze the FtsZ GTPase protein, inducing premature cell death. Huang et al. demonstrated the antibacterial properties of curcumin-encapsulated silver-polymeric NPs against *S. aureus* and *P. aeruginosa* [102].

Similarly, curcumin-capped micelles enhanced the miltefosine and alkylphosphocholine erufosine antibacterial properties against *S. aureus* [103]. The antiviral effects of curcumin micelles on hepatitis C virus (HCV) attachment were also demonstrated, where HCV cells treated with the nanocomplex had a lower viral load [104]. Similar results were observed in cells infected with the respiratory syncytial virus (RSV) when treated with curcumin-capped silver NPs [105].

Curcumin is a classical phenolic antioxidant which traps free radicals such as reactive nitrogen and oxygen species by enhancing the production of free radical scavenging enzymes [106,107,108,109,110,111]. Rajasekar (2015) demonstrated the antioxidant properties of curcumin nanocrystals in Wistar rats [108]. Nanocurcumin structures were observed to counteract the toxic side effects of aluminum phosphide by enhancing the stabilization of oxidative stress and scavenging free radicals [13].

Curcumin is a pleiotropic bioactive component which interacts with many inflammatory targets, including interleukins (IL) 1, 2, 5, 6, 8, and 12, macrophage inflammatory proteins (MIP), TNF-α (tumor necrosis factor-α), and the monocyte chemoattractant protein (MCP) [93,109,112,113]. The bioactive component hinders essential inflammatory response mediators, proinflammatory leukotrienes, and prostaglandin synthesis by downregulating the activity of lipoxygenase (LOX), cyclooxygenase-2 (COX-2), nitric oxide synthase (iNOS), and phospholipase A2 (PLA2) [101,110]. Thus, curcumin is effective in reducing post-surgical inflammation. The inflammatory response of curcumin can be attributed to its association with the arachidonic acid pathway for eicosanoid biosynthesis through the downregulation of COX-2 and LOX [95]. This produces many lipids, including prostaglandins, leukotrienes, prostacyclins, and thromboxanes. Nahar et al. (2015) demonstrated the ability of curcumin-capped lipid NPs to hinder the activation of NF-κB in murine macrophages through the downregulation of lipopolysaccharide-induced proinflammatory mediators, including IL-6, NO, and PGE2 [104,114]. Similarly, the obstruction of NF-κβ activation by curcumin-loaded PLGA NPs downregulated iNOS and COX-2 expression [103,115].

### 5.2. The Anticancer Properties of Curcumin

**Figure 8:**
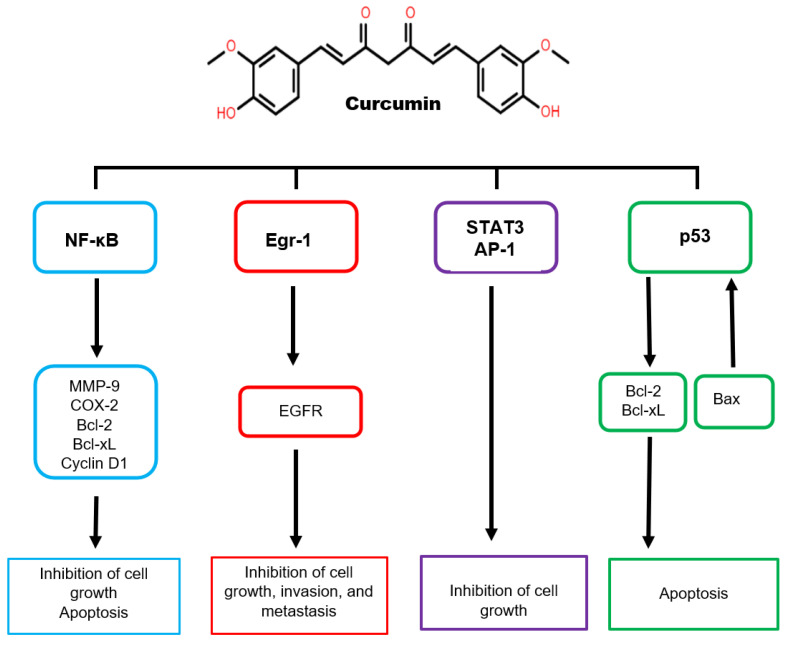
Antitumor effect of curcumin on the signaling pathways in cancer.

The anticancer properties of curcumin in humans have gained significant interest in the past few decades. Kuttan et al. (1987) initially reported the anticancer properties of curcumin in clinical trials on patients with external cancer lesions. The study revealed that curcumin effectively relieved the symptoms of pain and itching while decreasing the lesion size [113]. Curcumin has anticancer activities that alter various cell growth cycle stages [114]. It acts as a blocking agent, hindering the initial stages of cancer by suppressing the proliferation of malignant cells during carcinogenesis. The bioactive component acts on transcription factors, oncogenes, and signaling proteins, which facilitate cancer cells’ growth and metastasis at different carcinogenesis stages [105] (Figure 8). Curcumin can also suppress matrix metalloproteinases’ activity, thus averting cancer metastasis. This results from curcumin suppressing the Bcl-xL, nuclear factor-kappa (NF-κB), BCL-2, cyclin D1, and c-MYC genes involved in tumor proliferation, growth, and apoptosis [115]. Furthermore, curcumin downregulates mitogen and epidermal growth factor receptor-activated protein kinases in lung and pancreatic cancer cells. It also displayed anti-amyloid activity, which reduced β-secretase and acetylcholinesterase activity as well as amyloid-β-protein aggregation and inflammation [116].

Curcumin is a potential anticancer agent against several cancers, including thyroid, prostate, lung, liver, myeloma, pancreatic, melanoma, colorectal, breast, and cervical cancer. Curcumin NPs showed anticancer properties in skin, lung, and liver cancer cells [117]. PLGA-curcumin NPs enhanced apoptosis and lysosomal activity and deregulated nuclear β-catenin and androgen receptor (AR) activity in prostate cancer cells [118]. An in vitro study reported the ability of curcumin to inhibit the metastasis of the papillary thyroid tumor cells by regulating the expression pattern of E-cadherins and metalloproteinase-9 to enhance mesenchymal-epithelial transition [94]. An increase in HIF-1α, nuclear p65, and NF-κB expression was also noted, which altered the carcinogenic activity in breast cancer cells [119,120]. In vitro studies revealed enhanced the cytotoxicity and anti-invasive, anti-migratory, and apoptosis properties induced by curcumin-loaded NPs in metastatic pancreatic cancer [121]. The ability of curcumin nano-formulations to overcome the limitations of conventual treatment in colorectal cancer has been described in the literature [19].

## 6. Nanocurcumin Synthesis

Curcumin controls various signaling molecules based on the cell and target background, thus allowing it to act on multiple targets in cellular pathways [122]. However, the bioactive component has low bioavailability due to its limited solubility in water and its crystalline form [105]. To circumvent these constraints, researchers have attempted to increase curcumin’s biological and pharmacological potency by reducing its size [108]. This led to the discovery of nanocurcumin, which enhances the biological activity of curcumin, increasing its bioavailability, solubility, long-time circulation, and retention in the body [123].

The chemical and physical properties are essential in altering curcumin into its nanoform. The hydrophobicity, particle size, surface area, and charge are important physicochemical properties that make nanocurcumin a more effective anticancer agent than its native form [124]. The size of nanocurcumin being 1–100 nm is considered an ideal choice to use as a therapeutic agent because it has a larger surface area for better contact with the solvent. This enhances its solubility properties. Nanocurcumin structures can enter organs that are inaccessible to native curcumin [125]. Furthermore, nanocurcumin may have a higher intracellular absorption capacity, allowing the bioactive component to target foreign entities [126]. Zou et al. (2015) noted the high systemic bioavailability in the plasma and tissues of nanostructured curcumin compared with free curcumin [127]. Moreover, nanocurcumin increases the in vivo bioavailability and distribution, increasing the biological half-life 60-fold compared with treatment with native curcumin [93]. The loading and entrapment efficiency of nanodrugs depend highly on the preparation method and type of carrier system used to produce nanodrugs [128].

**Figure 9:**
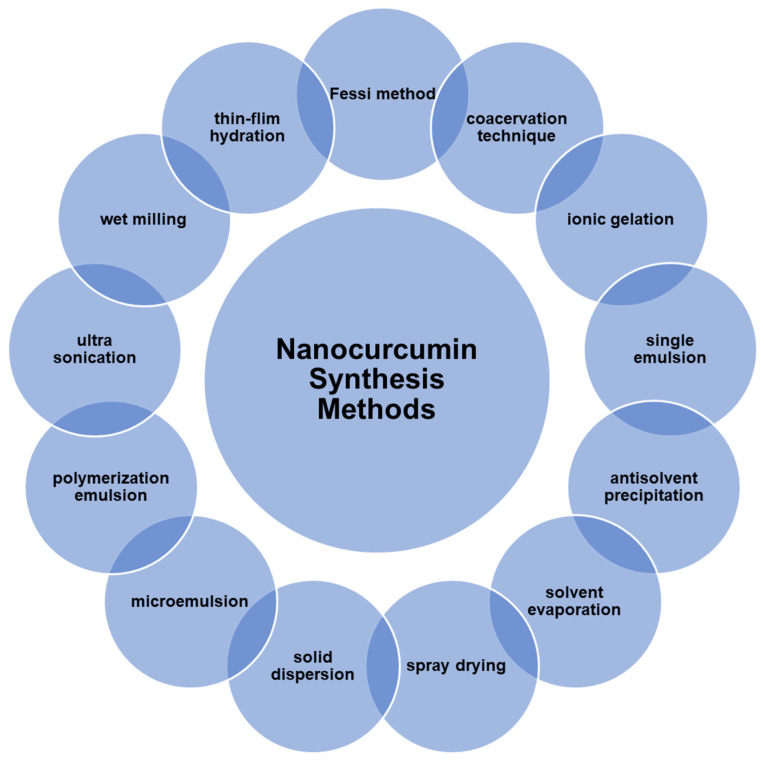
A summary of the methods used in nanocurcumin synthesis.

Nanocurcumin has been synthesized in various ways, including spray drying, emulsion polymerization, microemulsion, antisolvent precipitation, ultra-sonication, ionic gelation, single emulsion, solvent evaporation, wet milling, solid dispersion, thin film hydration, the Fessi method, and the coacervation technique (Figure 9). Each technique has its advantages and disadvantages, which have reviewed by many researchers [94,129,130].

The antisolvent precipitation and ionic gelation methods are regarded as the most efficient of the curcumin nano-formulation techniques [93].

These different techniques determine the shape and size of the curcumin NPs. Mukerjee and Vishwanatha (2009) reported the synthesis of 30–50-nm nanosphere curcumin structures using the polymerization emulsion technique [131]. Other studies reported the synthesis of crystal structured curcumin (150–200 nm) by the single emulsion–solvent evaporation method [132], small clusters (50 nm) using the inclusion complexation method [133], nanospheres (132 nm) using the redox-free radical polymerization technique [134], and nanocrystal curcumin NPs (30–40 nm) using the nanoprecipitation method [135]. Hence, it is evident that each technique can produce nanocurcumin of a defined size or shape. Based on these studies, researchers looking to synthesize curcumin NPs for biomedical applications would need to examine the size limitations of the target tissue or organ and the optimal shape of the NP for cellular uptake before opting for a particular synthesis method.

Nanocurcumin particles are not tissue-specific and act on healthy tissues surrounding the TME and cancer cells. Therefore, future studies must develop nano-delivery systems targeted at specific tissues [93]. Overall, the therapeutic effect of nanocurcumin remains at the concept level. Several questions and challenges prevail before nanocurcumin can be recommended as a promising candidate for therapeutic applications [136].

## 7. Curcumin-Capped NPs in Cancer Therapy

Curcumin can effectively reduce metal salts and cap the metal NPs. Curcumin-capped metal NPs have exhibited potent cytotoxicity in cancer cells [13]. Encapsulating therapeutic agents within NPs can enhance their pharmacokinetics and provide targeted delivery and controlled release [17]. Curcumin-capped NPs have a relatively larger surface area to interact with the solvent than naked curcumin. This property improves their aqueous solubility, leading to better bioavailability of the bound or encapsulated therapeutic. This enhances the therapeutic’s response to a specific molecular target and improves its pharmacological activity [137] by promoting controlled drug release [138]. Although nanocurcumin has a higher water solubility than curcumin-capped NPs, reducing and capping metal NPs with curcumin prevents their aggregation, making them highly stable in a solution [139,140,141,142,143]. This good stability and the ability of curcumin-capped NPs to be easily dissolved in an aqueous solution enhances their cellular internalization [8].

To date, solid lipid, polymeric, magnetic, and gold-based NPs have all been employed to enhance curcumin’s therapeutic application (Table 2).

The antioxidant property of curcumin facilitates the reduction of metal salts by transferring electrons from curcumin to metal ions. The functional carbonyl and hydroxyl groups of the free curcumin further stabilize the NPs [126,127,128,129]. Solid lipid NPs are colloidal particles of natural or synthetic lipids. They are stable, easily scalable, and display enhanced biocompatibility, further improving solubility [140]. Solid lipid NPs conjugated with curcumin enhanced the therapeutic agent’s solubility, cellular uptake, dispersibility, and stability [111]. Wang et al. (2013) demonstrated that curcumin had increased inhibition (from 19.5% to 69.3%) and enhanced apoptosis in lung cancer cells both in vitro and in vivo [140].

The biocompatibility and compact size of polymeric NPs enable them to circulate within the blood system for an extended period. Chaurasia et al. (2016) observed the ability of a Eudragit R E100 cationic copolymer to enhance the uptake, binding, and cytotoxicity of curcumin-conjugated polymeric NPs in colon-26 cells [142].

The chemical properties of magnetic NPs, formed from a metallic oxide core, are easily manipulated regarding shape and size. Furthermore, these low-cost magnetic NPs display unique physical properties, promoting their biocompatibility in the human body [38]. PEGylated magnetic NPs conjugated with curcumin displayed enhanced biocompatible and antitumor responses [147]. The sustainable delivery of curcumin-loaded, thiolated, starch-capped iron-oxide NPs to lymphocyte cells, inducing cytotoxicity in several cancer cell lines, was demonstrated. Curcumin-capped Fe_3_O_4_-magnetic NPs illustrated enhanced uptake and targeted drug delivery to tumor cells [148].

Gold is a valuable metal, prized for its economic value and aesthetic appeal. Gold NPs serve as potential therapeutic gene delivery vehicles due to their favorable properties and ease of manufacturing [149]. Furthermore, due to their tunable stability, resilience, biodegradability, low cytotoxicity, biocompatibility, therapeutic gene protection from systemic degradation, and synthetic surface amenability, gold NPs have been used in various biomedical applications [46,150,151]. Targeted delivery of therapeutic agents and enhanced apoptosis in colon tumor cells was demonstrated using curcumin-reduced and capped chitosan-gold NPs [152]. Curcumin-capped gold NPs also effectively induced apoptosis in prostate and renal cancer cells [127,153]. Furthermore, green synthesized curcumin-capped gold NPs were found to improve antiproliferative and apoptotic activities in breast (MCF-7) and colon (HCT-116) cancer cells [126]. Gold NPs capped with curcumin and folic acid were observed to decrease tumor proliferation in breast cancer cells of Balb/c mice by 51% [154].

## 8. Clinical Trials Involving Curcumin

Numerous clinical trials have explained curcumin’s pharmacokinetic profile, safety, and effectiveness in different diseases. Clinical trials showed positive results where curcumin arrested or even eliminated cancer cell growth [6,137,139]. A search for clinical trials using curcumin or curcumin-reduced and capped NPs revealed that no curcumin-capped NPs are currently being screened. Most trials involved the delivery of curcumin alone or in combination with another therapeutic. Only one study utilized albumin NPs in combination with curcumin for pancreatic cancer therapy. The pilot phase I trial study demonstrated the ability of the nanotherapeutic agent to slow down cancer growth by halting tumor cell division. Table 3 provides a summary of the clinical trials involving curcumin [155]. Trials that have been withdrawn or terminated are not reflected.

## 9. Conclusions and Future Perspectives

Green synthesized NPs have revolutionized nanotechnology. The NPs work synergistically with the conjugated plant extract, enhancing anticancer activity and biocompatibility. Using green nanotechnology to treat cancer provides the exciting prospect of inducing apoptosis while causing minimal damage to healthy cells surrounding the TME. Due to the toxicity challenges associated with chemical synthesis, the possibility of novel green synthesis methods emerging to overcome this problem is imminent. More research is needed to fully understand the various mechanisms involved in reducing organic and inorganic salts to produce NPs by the various green synthesis techniques. Green nanotechnology still has many unknowns to unravel and much to accomplish. Plants especially contain bioactive molecules that can act as reducing agents in NP synthesis. One such compound is curcumin.

Curcumin, due to its range of biomedical properties, including anticancer activity, has attracted the attention of researchers. Curcumin’s limited solubility in water, crystalline form, and low bioavailability have led to the formulation of nanocurcumin. Nanocurcumin enhances the cellular uptake, antitumor properties, tissue specificity, and pharmacokinetics of the conjugated therapeutic agent. The synthesis of inorganic and metal NPs by curcumin reduction especially has yet to be taken advantage of. This reduction process leads to the curcumin capping of the NPs, imparting synergistic activity with the therapeutic cargo. Optimizing synthesis protocols for the further application of curcumin capped-NPs is necessary to ensure these NPs are inexpensive, non-toxic, and can be formulated on a large scale for commercial use. Great potential lies in the curcumin reduction of metal salts to produce metal NPs such as gold and silver, which are commonly chemically synthesized, and improve their potential as delivery vehicles in cancer therapy. Comparative studies of chemical versus green synthesis need to be undertaken before definitive conclusions can be made. The crucial properties such as stability, toxicity, size, and shape must be optimized. The challenges of controlling the size and shape using curcumin or other green synthesis methods must be overcome.

Most of the research to date has been carried out in preclinical models at the proof-of-concept stage. Overall, there is still a significant dearth of knowledge about the full impact of curcumin and its capped NPs and their long-term risks in humans. Hence, before introducing curcumin-synthesized nano-formulations to the pharmaceutical sector, more in vitro and in vivo studies are required before clinical trials can be undertaken.

## Figures and Tables

**Table 1:** Summary of nanoparticle synthesis methods and their advantages and disadvantages.

Synthesis	Advantages	Disadvantages	Types
Physical	- Simple procedure - Produced in large quantities - Controlled particle interspacing	- High energy consumption - Expensive - Specialized equipment required	Radiation Sonication Laser ablation Membrane filtration Ion exchange
Chemical	- Size, shape, and morphology controlled - Simple procedure - Narrow size distribution	- Toxic chemicals - Long reaction time - Limited by external factors (pH and temperature)	Reduction Oxidative process Photochemical Electrochemical destruction Condensation Sol-gel method
Biological	- Eco-friendly - Use of non-toxic chemicals - Inexpensive - Less energy required	- Degree of reducibility - Reducing extract needs to be elucidated - Limited knowledge of controlled shape and size	Plants Bacteria Fungi Viruses

**Table 2:** Curcumin-conjugated nanoparticles and their anticancer activities.

Curcumin-Conjugate		Morphology		Cancer Model	Anticancer Activity	Ref
Nanoparticle (Source)		Shape	Size (nm)			
Solid lipid (Stearic acid and lecithin)	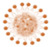	Spherical	20–80	Breast cancer	Increased Bax/Bcl-2 ratios. Enhanced biocompatibility.	[140]
Micelles (d-hydroxyethyl starch)	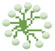	Spherical	34.9	Lung and colorectal cancer	Improved bioavailability, blood circulation, solubility, and stability of the nanocomplex. Enhanced antiproliferation and apoptosis.	[129]
Liposomes (l-α-phosphatidyl choline and cholesterol)	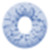	Irregular spherical	10–50	Melanoma and Lung cancer	Greater encapsulation activity. Enhanced antiproliferation and apoptosis.	[137]
Polymeric (Poloxamer 188)	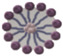	Spherical	248.4	Colon and ovarian carcinoma	Enhanced uptake and cell specificity. Increased cytotoxicity. Improved blood circulation.	[143]
Silver (Silver nitrate)	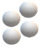	Spherical	15.5	Breast cancer	Enhanced cellular uptake. Enhanced antiproliferation and apoptosis.	[141]
Gold (Chloroauric acid)	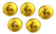	Spherical	26–28.2	Breast, colon, prostate, and renal carcinoma	Improved blood circulation, solubility, and stability of the nanocomplex. Enhanced antiproliferation and apoptosis.	[140,142,144]
Albumin (Bovine serum albumin)	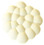	Spherical	112–198	Breast cancer	Enhanced antiproliferation and apoptosis.	[145]
Graphene oxide and quantum dots (Graphite powder)	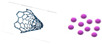	Crystal-like	15.62	Breast cancer	Enhanced cellular uptake. Increased cytotoxicity.	[146]

**Table 3:** Selected completed or ongoing clinical trials from 2004 to date utilizing curcumin in cancer therapy (adapted from [155]).

Cancer	Study Title	Therapeutic	Start and End Dates
Breast	“Window Trial” on Curcumin for Invasive Breast Cancer Primary Tumors	* Curcumin	January 2020– December 2022
	Curcumin in Reducing Joint Pain in Breast Cancer Survivors with Aromatase inhibitor-induced Joint Disease	** Curcumin Nanoemulsion	March 2019– July 2022
	# Curcumin for the Prevention of Radiation-induced Dermatitis in Breast Cancer Patients	* Curcumin c3	January 2008–April 2011
	# Pilot Study of Curcumin for Women with Obesity and High Risk for Breast Cancer	* Curcumin	June 2013– September 2016
	# Phase II Study of Curcumin vs Placebo for Chemotherapy-Treated Breast Cancer Patients Undergoing Radiotherapy	* Curcumin	May 2015– July 2018
	# Prophylactic Topical Agents in Reducing Radiation-Induced Dermatitis in patients With Non-inflammatory Breast Cancer	* Curcumin	October 2015–September 2016
	# Curcumin in Combination with Chemotherapy in Advanced Breast Cancer	** Curcumin, Paclitaxel	March 2017– June 2019
	# Disposition of Dietary Polyphenols and Methylxanthines in Mammary Tissues from Breast Cancer Patients	** Curcumin Polyphenol	June 2017– December 2019
Colon	Study Investigating the Ability of Plant Exosomes to Deliver Curcumin to Normal and Colon Cancer Tissue	* Curcumin	January 2011– December 2022
	# Curcumin Biomarkers	* Curcumin c3	November 2010–January 2013
	# Combining Curcumin with FOLFOX Chemotherapy in Patients with inoperable Colorectal Cancer	** Curcumin Chemotherapy	February 2012–May 2017
	# Effect of Curcumin on Dose Limiting Toxicity and Pharmacokinetics of Irinotecan in Patients with Solid Tumors	** Curcumin, Irinotecan	June 2013– October 2016
	# Avastin/FOLFIRI in Combination with Curcumin in Colorectal Cancer Patients with Unresectable Metastasis	** Curcumin Avastin/FOLFIRI	August 2015–2019
Cervical	Curcumin in Advanced Cervical Cancer	* Curcumin	December 2021–2023
	# Trial on Safety and Pharmacokinetics of Intravaginal Curcumin	* Curcumin	January 2010–2012
	# Study of Pembrolizumab, Radiation and Immune Modulatory Cocktail in Cervical/Uterine Cancer	** Curcumin, Pembrolizumab Radiation, Vitamin D Aspirin, Lansoprazole Cyclophosphamide	July 2017– June 2021
Prostate	Adjuvant Curcumin to Assess Recurrence-Free Survival in Patients Who Have Had a Radical Prostatectomy	* Curcumin	May 2014– June 2023
	Trial of Curcumin to Prevent Progression of Low-risk Prostate Cancer Under Active Surveillance	* Curcumin	March 2016– November 2026
	Curcumin and Piperine in Patients on Surveillance for Monoclonal Gammopathy, Smoldering Myeloma or Prostate Cancer	** Curcumin, Piperine	December 2021–May 2023
	# Comparison of Duration of Treatment Interruption with or Without Curcumin During the off-Treatment Periods in Patients with Prostate Cancer Undergoing Intermittent Androgen Deprivation Therapy	* Curcumin	August 2007–2015
	# Radiosensitizing and Radioprotective Effects of Curcumin in Prostate Cancer	* Curcumin	March 2011– October 2019
	# Multicentre International Study for the Prevention with Ialuril® of Radio-induced Cystitis (MISTIC)	** Curcumin Radiotherapy	April 2017– May 2019
	# Correlative Analysis of the Genomics of Vitamin D and Omega-3 Fatty Acid Intake in Prostate Cancer	** Curcumin Vitamin D, Omega-3	September 2017–December 2019
Lung	Phase II Trial to Modulate Intermediate Endpoint Biomarkers in Former and Current Smokers	** Curcumin, Lovaza	June 2019– October 2023
	The Thoracic Peri-Operative Integrative Surgical Care Evaluation Trial-Stage II	** Curcumin, Vitamin D3 Coriolus Versicolor Provitalix Green Tea Extract	April 2022– May 2025
Head and Neck	# Curcumin Biomarker Trial in Head and Neck Cancer	* Curcumin c3	June 2010– January 2016
	# Curcumin Bioavailability in Glioblastoma Patients	* Curcumin	October 2012–May 2013
	# The Effect of Curcumin on Treatment of Cancer Anorexia-Cachexia Syndrome in Patients with Stage III-IV of Head and Neck Cancer	* Curcumin	February 2020–March 2021
Leukaemia	Safety and Efficacy of Curcumin in Children with Acute Lymphoblastic Leukemia	* Curcumin	August 2021–September 2022
Oral	# Oral Curcumin for Radiation Dermatitis	* Curcumin	February 2011–January 2015
Pancreatic	Gemcitabine Hydrochloride, Paclitaxel Albumin- Stabilized Nanoparticle Formulation, Metformin Hydrochloride, and a Standardized Dietary Supplement in Treating Patients with Pancreatic Cancer That Cannot Be Removed by Surgery	** Curcumin Gemcitabine Albumin Metformin	January 2016– December 2022
	# Gemcitabine With Curcumin for Pancreatic Cancer	** Curcumin, Gemcitabine	July 2004– September 2010
	# Trial of Curcumin in Advanced Pancreatic Cancer	* Curcumin	November 2004–April 2014

* Single therapy. ** Dual therapy. # Completed trials.

## Data Availability

Not applicable.

## References

[1] Krasteva N., Georgieva M. (2022). Promising Therapeutic Strategies for Colorectal Cancer Treatment Based on Nanomaterials. Pharmaceutics.

[2] Venkatas J., Singh M. (2021). Nanomedicine-mediated optimization of immunotherapeutic approaches in cervical cancer. Nanomedicine.

[3] Sung H., Ferlay J., Siegel R.L., Laversanne M., Soerjomataram I., Jemal A., Bray F. (2021). Global Cancer Statistics 2020: GLOBOCAN Estimates of Incidence and Mortality Worldwide for 36 Cancers in 185 Countries. CA Cancer J. Clin..

[4] Ward Z.J., Scott A.M., Hricak H., Atun R. (2021). Global costs, health benefits, and economic benefits of scaling up treatment and imaging modalities for the survival of 11 cancers: A simulation-based analysis. Lancet Oncol..

[5] Venkatas J., Singh M. (2020). Cervical cancer: A meta-analysis, therapy, and future of nanomedicine. Ecancermedicalscience.

[6] Juárez A.A.S., Alvarado E.M., Gallegos E.R. (2019). Cell death induced by photodynamic therapy with the conjugate of gold nanoparticles-PpIX in HeLa cell line. AIP Conf. Proc..

[7] Sun Q., Barz M., De Geest B.G., Diken M., Hennink W.E., Kiessling F., Lammers T., Shi Y. (2019). Nanomedicine and macroscale materials in immuno-oncology. Chem. Soc. Rev..

[8] Muniyappan N., Pandeeswaran M., Amalraj A. (2021). Green synthesis of gold nanoparticles using Curcuma pseudomontana isolated curcumin: Its characterization, antimicrobial, antioxidant, and anti-inflammatory activities. Environ. Chem. Ecotoxicol..

[9] Rajput N. (2015). Methods of preparation of nanoparticles-a review. Int. J. Adv. Eng. Technol..

[10] Menon S., Shanmugam R., Kumar V. (2017). A review on biogenic synthesis of gold nanoparticles, characterization, and its applications. Resour. Effic. Technol..

[11] Olawale F., Oladimeji O., Ariatti M., Singh M. (2022). Emerging Roles of green synthesized Chalcogen and Chalcogenide nanoparticles in Cancer theranostics. J. Nanotechnol..

[12] Kuppusamy P., Yusoff M., Maniam G.P., Govindan N. (2016). Biosynthesis of metallic nanoparticles using plant derivatives and their new avenues in pharmacological applications-an updated report. Saudi Pharm. J..

[13] Ranjbar A., Gholami L., Ghasemi H., Kheiripour N. (2020). Effects of nano-curcumin and curcumin on the oxidant and antioxidant system of the liver mitochondria in aluminum phosphide-induced experimental toxicity. Nanomed. J..

[14] Saranya T.S., Rajan V.K., Biswas R., Jayakumar R., Sathianarayanan S. (2018). Synthesis, characterization, and biomedical applications of curcumin conjugated chitosan microspheres. Int. J. Biol. Macromol..

[15] Shen L., Liu C.-C., An C.-Y., Ji H.-F. (2016). How does curcumin work with poor bioavailability? Clues from experimental and theoretical studies. Sci. Rep..

[16] Ubeyitogullari A., Ciftci O.N. (2019). A novel and green nanoparticle formation approach to forming low-crystallinity curcumin nanoparticles to improve curcumin’s bioaccessibility. Sci. Rep..

[17] Chen S., Wu J., Tang Q., Xu C., Huang Y., Huang D., Wang S. (2020). Nano-micelles based on hydroxyethyl starch-curcumin conjugates improve curcumin’s stability, antioxidant, and anticancer activity. Carbohydr. Polym..

[18] Al Bostami R.D., Abuwatfa W.H., Husseini G.A. (2022). Recent Advances in Nanoparticle-Based Co-Delivery Systems for Cancer Therapy. Nanomaterials.

[19] Wong K.E., Ngai S.C., Chan K.G., Lee L.H., Goh B.H., Chuah L.H. (2019). Curcumin nanoformulations for colorectal cancer: A review. Front. Pharmacol..

[20] World Health Organization (WHO) (2021). Global Health Estimates 2020: Deaths by Cause, Age, Sex, by Country and by Region, 2000–2020. https://gco.iarc.fr/today/home.

[21] Lin L., Yan L., Liu Y., Yuan F., Li H., Ni J. (2019). Incidence, and death in 29 cancer groups in 2017 and trend analysis from 1990 to 2017 from the Global Burden of Disease Study. J. Hematol. Oncol..

[22] Rivenbark A.G., Coleman W.E., Tsongalis G.J. (2017). An Overview of Cancer Genes. The Molecular Basis of Human Cancer.

[23] Padayachee J., Daniels A.N., Balgobind A., Ariatti M., Singh M. (2020). HER-2/neu and MYC gene silencing in breast cancer: Therapeutic potential and advancement in non-viral nanocarrier systems. Nanomedicine.

[24] Momenimovahed Z., Salehiniya H. (2017). Incidence, mortality, and risk factors of cervical cancer in the world. Biomed. Res. Ther..

[25] Maistro S., Teixeira N., Encinas G., Katayama M.L.H., Niewiadonski V.D.T., Cabral L.G., Sabino E.C. (2016). Germline mutations in BRCA1 and BRCA2 in epithelial ovarian cancer patients in Brazil. BMC Cancer.

[26] Sample K.M. (2020). DNA repair gene expression is associated with differential prognosis between HPV16 and HPV18 positive cervical cancer patients following radiation therapy. Sci. Rep..

[27] Chen K., Husain S., Marathe A., Haq M. (2018). Molecular Genetics of Cancer. Int. J. Hum. Health Sci..

[28] Roy N.K., Bordoloi D., Monisha J., Anip A., Padmavathi G., Kunnumakkara A.B., Kunnumakkara A.B., Padmavathi G., Roy N.K. (2017). Cancer—An Overview and Molecular Alterations in Cancer. Fusion Genes and Cancer.

[29] Rajabi M., Mousa S.A. (2017). The role of angiogenesis in cancer treatment. Biomedicines.

[30] Tomasetti C., Li L., Vogelstein B. (2017). Stem cell divisions, somatic mutations, cancer etiology, and cancer prevention. Science.

[31] Chaturvedi P., Singh A., Chien C.Y., Warnakulasuriya S. (2019). Tobacco related oral cancer. BMJ.

[32] Miura K., Olsen C.M., Rea S., Marsden J., Green A.C. (2019). Melanoma and skin cancers in airline pilots and cabin crew. Br. J. Dermatol..

[33] Murtono M., Ndii M.Z., Sugiyanto S. (2019). Mathematical model of cervical cancer treatment using chemotherapy drug. Biol. Med. Nat. Prod. Chem..

[34] Kong S.Y., Huang K., Zeng C., Ma X., Wang S. (2018). The association between short-term response and long-term survival for cervical cancer patients undergoing neoadjuvant chemotherapy: A system review and meta-analysis. Sci. Rep..

[35] Lohitesh K., Chowdhury R., Mukherjee S. (2018). Resistance a major hindrance to chemotherapy in hepatocellular carcinoma: An insight. Cancer Cell Int..

[36] Feynman R.P. (1960). There’s Plenty of Room at the Bottom. Eng. Sci..

[37] Soares S., Sousa J., Pais A., Vitorino C. (2018). Nanomedicine: Principles, properties, and regulatory issues. Front. Chem..

[38] Roacho-Pérez J.A., Ruiz-Hernandez F.G., Chapa-Gonzalez C., Martínez-Rodríguez H.G., Flores-Urquizo I.A., Pedroza-Montoya F.E., Garza-Treviño E.N., Bautista-Villareal M., García-Casillas P.E., Sánchez-Domínguez C.N. (2020). Magnetite Nanoparticles Coated with PEG 3350-Tween 80: *In vitro* Characterization Using Primary Cell Cultures. Polymers.

[39] Habib S., Singh M. (2021). Recent advances in lipid-based nanosystems for gemcitabine and gemcitabine–combination therapy. Nanomaterials.

[40] Cao J., Huang D., Peppas N.A. (2020). Advanced engineered nanoparticulate platforms to address key biological barriers for delivering chemotherapeutic agents to target sites. Adv. Drug Deliv. Rev..

[41] El-Readi M.Z., Althubiti M.A. (2019). Cancer nanomedicine: A new era of successful targeted therapy. J. Nanomater..

[42] Oladimeji O., Akinyelu J., Daniels A., Singh M. (2021). Modified Gold Nanoparticles for efficient Delivery of Betulinic Acid to Cancer Cell Mitochondria. Int. J. Mol. Sci..

[43] Joseph C., Daniels A., Singh S., Singh M. (2022). Histidine-tagged Folate-Targeted Gold Nanoparticles for enhanced transgene expression in Breast Cancer Cells in vitro. Pharmaceutics.

[44] Maiyo F., Singh M. (2020). Polymerized Selenium nanoparticles for Folate-Receptor Targeted Delivery of anti-Luc-siRNA: Potential for Gene Silencing. Biomedicines.

[45] Moitra K. (2015). Overcoming multidrug resistance in cancer stem cells. Bio. Med. Res. Int..

[46] Li B., Lane L.A. (2019). Probing the biological obstacles of nanomedicine with gold nanoparticles. Wiley Interdiscip. Rev. Nanomed. Nanobiotechnol..

[47] Naicker K., Ariatti M., Singh M. (2016). Active targeting of asiaglycoprotein receptors using sterically stabilized lipoplexes. Eur. J. Lipid Sci. Technol..

[48] Ragelle H., Danhier F., Préat V., Langer R., Anderson D.G. (2017). Nanoparticle-based drug delivery systems: A commercial and regulatory outlook as the field matures. Exp. Opin. Drug Deliv..

[49] Moodley T., Singh M. (2020). Sterically Stabilized Polymeric Mesoporous Silica Nanoparticles Improve Doxorubicin Efficiency: Tailored Cancer Therapy. Molecules.

[50] Daniels A., Singh M., Ariatti M. (2013). Pegylated and Non-Pegylated siRNA lipoplexes formulated with cholesteryl cytofectins promote efficient Luciferase knockdown in HeLa tat luc cells. Nucleos. Nucleot. Nucl..

[51] Omidi Y., Barar J. (2014). Targeting tumor microenvironment: Crossing tumor interstitial fluid by multifunctional nanomedicines. BioImpacts B.

[52] Shanker U., Jassal V., Rani M., Kaith B.S. (2016). Towards Green Synthesis of Nanoparticles: From Bio-Assisted Sources to Benign Solvents. A Review. Int. J. Environ. Anal. Chem..

[53] Srivastava S., Usmani Z., Atanasov A.G., Singh V.K., Singh N.P., Abdel-Azeem A.M., Prasad R., Gupta G., Sharma M., Bhargava A. (2021). Biological nanofactories: Using living forms for metal nanoparticle synthesis. Mini Rev. Med. Chem..

[54] Peralta-Videa J.R., Huang Y., Parsons J.G. (2016). Plant-based green synthesis of metallic nanoparticles: Scientific curiosity or a realistic alternative to chemical synthesis?. Nanotechnol. Environ. Eng..

[55] Rafique M., Tahir R., Gillani S.S.A., Tahir M.B., Shakil M., Iqbal T., Abdellahi M.O. (2022). Plant-mediated green synthesis of zinc oxide nanoparticles from Syzygium Cumini for seed germination and wastewater purification. Int. J. Environ. Anal. Chem..

[56] Guan Z., Ying S., Ofoegbu P.C., Clubb P., Rico C., He F., Hong J. (2022). Green synthesis of nanoparticles: Current developments and limitations. Environ. Technol. Innov..

[57] Kumari S.C., Dhand V., Padma N., Kumar R.P., Bharathiraja B. (2021). Green synthesis of metallic nanoparticles: A review. Nanomaterials, Application in Biofuels and Bioenergy Production Systems.

[58] Dahoumane S.A., Yéprémian C., Djédiat C. (2016). Improvement of kinetics, yield, and colloidal stability of biogenic gold nanoparticles using living cells of Euglena gracilis microalga. J. Nanoparticl. Res..

[59] Khan M., Al-Marri A.H., Khan M. (2015). Green approach for the effective reduction of graphene oxide using *Salvadora persica* L. root (Miswak) extract. Nanoscale Res Lett..

[60] Hano C., Abbasi B.H. (2021). Plant-Based Green Synthesis of Nanoparticles: Production, Characterization and Applications. Biomolecules.

[61] Ijaz I., Gilani E., Nazir A., Bukhari A. (2020). Detail review on chemical, physical and green synthesis, classification, characterizations, and applications of nanoparticles. Green Chem. Lett. Rev..

[62] Banach M., NPulit-Prociak J. (2017). Proecological method for the preparation of metal nanoparticles. J. Clean. Prod..

[63] Pedroza-Toscano M.A., Rabelero-Velasco M., Díaz de León R., Saade H., López R.G., Mendizábal E., Puig J.E. (2012). Preparation of silver nanostructures from bicontinuous microemulsions. J. Nanomater..

[64] Rashid M.U., Bhuiyan M.K.H., Quayum M.E. (2013). Synthesis of silver nanoparticles (Ag-NPs) and their uses for quantitative analysis of vitamin C tablets. Dhaka Univ. J. Pharm. Sci..

[65] Tsekhmistrenko S.I., Bityutskyy V.S., Tsekhmistrenko O.S., Horalskyi L.P., Tymoshok N.O., Spivak M.Y. (2020). Bacterial synthesis of nanoparticles: A green approach. Biosyst. Divers..

[66] Mareeswari P., Brijitta J., Etti S.H., Meganathan C., Kaliaraj G.S. (2016). Rhizopus stolonifer mediated biosynthesis of biocompatible cadmium chalcogenide quantum dots. Enzyme Microb. Technol..

[67] Gholami-Shabani M., Shams-Ghahfarokhi M., Gholami-Shabani Z., Akbarzadeh A., Riazi G., Ajdari S., Amani A., Razzaghi-Abyaneh M. (2015). Enzymatic synthesis of gold nanoparticles using sulfite reductase purified from Escherichia coli: A green eco-friendly approach. Process Biochem..

[68] Li J., Tian B., Li T., Dai S., Weng Y., Lu J., Xu X., Jin Y., Pang R., Hua Y. (2018). Biosynthesis of Au, Ag and Au–Ag bimetallic nanoparticles using protein extracts of Deinococcus radiodurans and evaluation of their cytotoxicity. Int. J. Nanomed..

[69] Iranmanesh S., Bonjar G.H.S., Baghizadeh A. (2020). Study of the biosynthesis of gold nanoparticles by using several saprophytic fungi. SN Appl. Sci..

[70] Elshafei A.M., Othman A.M., Elsayed M.A., Al-Balakocy N.G., Hassan M.M. (2021). Green synthesis of silver nanoparticles using Aspergillus oryzae NRRL447 exogenous proteins: Optimization via central composite design, characterization, and biological applications. Environ. Nanotechnol. Monit. Manag..

[71] Dikshit P.K., Kumar J., Das A.K., Sadhu S., Sharma S., Singh S., Kim B.S. (2021). Green Synthesis of Metallic Nanoparticles: Applications and Limitations. Catalysts.

[72] Deepak P., Amutha V., Kamaraj C., Balasubramani G., Aiswarya D., Perumal P., Shukla A.K., Iravani S. (2019). Chemical and green synthesis of nanoparticles and their efficacy on cancer cells. Micro and Nanotechnologies, Green Synthesis, Characterization and Applications of Nanoparticles.

[73] Jadoun S., Arif R., Jangid N.K., Meena R.K. (2021). Green synthesis of nanoparticles using plant extracts: A review. Environ. Chem. Lett..

[74] Naidoo C.M., Naidoo Y., Dewir Y.H., Singh M., Daniels A.N., El-Ramady H. (2022). In vitro investigation of the antioxidant and cytotoxic potential of *Tabernaemontana ventricosa Hochst*. ex A. DC. leaf, stem, and latex extracts. Horticulturae.

[75] Patra D., El Kurdi R. (2021). Curcumin as a novel reducing and stabilizing agent for the green synthesis of metallic nanoparticles. Green Chem. Lett. Rev..

[76] Nahari M.H., Al Ali A., Asiri A., Mahnashi M.H., Shaikh I.A., Shettar A.K., Hoskeri J. (2022). Green Synthesis and Characterization of Iron Nanoparticles Synthesized from Aqueous Leaf Extract of *Vitex leucoxylon* and Its Biomedical Applications. Nanomaterials.

[77] Latif M.S., Abbas S., Kormin F., Mustafa M.K. (2019). Green synthesis of plant-mediated metal nanoparticles: The role of polyphenols. Asian J. Pharmaceut. Clin. Res..

[78] Fabiyi O.A., Alabi R.O., Ansari R.A., Ansari R., Rizvi R., Mahmood I. (2020). Nanoparticles’ synthesis and their application in the management of phytonematodes: An overview. Management of Phytonematodes: Recent Advances and Future Challenges.

[79] Azevedo de M. Oliveira L.F., de Azevedo Teles da Silva L.V., do Nascimento T.G., de Almeida L.M., Calumby R.J.N., Nunes Á.M., de Magalhães Oliveira L.M.T., da Silva Fonseca E.J. (2020). Antioxidant, and antimicrobial activity of red propolis embedded mesoporous silica nanoparticles. Drug Dev. Ind. Pharm..

[80] Sankar R., Maheswari R., Karthik S., Shivashangari K.S., Ravikumar V. (2014). Anticancer activity of *Ficus religiosa* engineered copper oxide nanoparticles. Mater. Sci. Eng. C.

[81] Dorosti N., Jamshidi F. (2016). Plant-mediated gold nanoparticles by Dracocephalum kotschyi as anticholinesterase agent: Synthesis, characterization, and evaluation of anticancer and antibacterial activity. J. Appl. Biomed..

[82] Muthukrishnan S., Kumar T.S., Rao M.V. (2017). Anticancer activity of biogenic nanosilver and its toxicity assessment on Artemia salina-evaluation of mortality, accumulation, and elimination: An experimental report. J. Environ. Chem. Eng..

[83] Seetharaman P., Chandrasekaran R., Gnanasekar S., Mani I., Sivaperumal S. (2017). Biogenic gold nanoparticles synthesized using Crescentia cujete L. and evaluation of their different biological activities. Biocatal. Agric. Biotechnol..

[84] Boomi P., Poorani G.P., Selvam S., Palanisamy S., Jegatheeswaran S., Anand K., Balakumar C., Premkumar K., Prabu H.G. (2020). Green biosynthesis of gold nanoparticles using Croton sparsiflorus leaves extract and evaluation of UV protection, antibacterial and anticancer applications. Appl. Organomet. Chem..

[85] Nayak D., Pradhan S., Ashe S., Rauta P.R., Nayak B. (2015). Biologically synthesized silver nanoparticles from three diverse family of plant extracts and their anticancer activity against epidermoid A431 carcinoma. J. Colloid Interface Sci..

[86] Venugopal K., Ahmad H., Manikandan E., Arul K.T., Kavitha K., Moodley M.K., Rajagopal K., Balabhaskar R., Bhaskar M. (2017). The impact of anticancer activity upon Beta vulgaris extract mediated biosynthesized silver nanoparticles (ag-NPs) against human breast (MCF-7), lung (A549) and pharynx (Hep-2) cancer cell lines. J. Photochem. Photobiol. B Biol..

[87] Olawale F., Ariatti M., Singh M. (2020). Ocimum tenuiflorum L. Mediated Green Synthesis of Silver and Selenium Nanoparticles: Antioxidant activity, Cytotoxicity and Density Functional Theory Studies. Adv. Nat. Sci. Nanosci. Nanotechnol..

[88] Olawale F., Ariatti M., Singh M. (2021). Biogenic Synthesis of Silver-Core Selenium-Shell Nanoparticles Using Ocimum tenuiflorum L.: Response Surface Methodology Based Optimization and Biological Activity. Nanomaterials.

[89] Naraginti S., Li Y. (2017). Preliminary investigation of catalytic, antioxidant, anticancer and bactericidal activity of green synthesized silver and gold nanoparticles using Actinidia deliciosa. J. Photochem. Photobiol. B Biol..

[90] Bello B.A., Khan S.A., Khan J.A., Syed F.Q., Mirza M.B., Shah L., Khan S.B. (2017). Anticancer, antibacterial and pollutant degradation potential of silver nanoparticles from Hyphaene thebaica. Biochem. Biophys. Res. Commun..

[91] Aygun A., Gülbagca F., Ozer L.Y., Ustaoglu B., Altunoglu Y.C., Celik Y., Baloglu M.C., Atalar M.N., Alma M.H., Sen F. (2020). Biogenic platinum nanoparticles using black cumin seed and their potential usage as antimicrobial and anticancer agent. J. Pharm. Biomed. Anal..

[92] Aswini R., Murugesan S., Kannan K. (2020). Bio-engineered TiO_2_ nanoparticles using Ledebouria revoluta extract: Larvicidal, histopathological, antibacterial, and anticancer activity. Int. J. Environ. Anal. Chem..

[93] Karthikeyan A., Senthil N., Min T. (2020). Nanocurcumin: A promising candidate for therapeutic applications. Front. Pharmacol..

[94] Rai M., Pandit R., Gaikwad S., Yadav A., Gade A. (2015). Potential applications of curcumin and curcumin nanoparticles: From traditional therapeutics to modern nanomedicine. Nanotechnol. Rev..

[95] Ahmad K., Ansari V.A., Singh K., Kushwaha P., Akhtar J. (2015). *Curcuma longa*: Boon for health care system with its biomedical application. Int. J. Pharm. Sci. Res..

[96] Amalraj A., Pius A., Gopi S., Gopi S. (2017). Biological activities of curcuminoids, other biomolecules from turmeric and their derivatives–A review. J. Tradit. Complement. Med..

[97] Nelson K.M., Dahlin J.L., Bisson J., Graham J., Pauli G.F., Walters M.A. (2017). The essential medicinal chemistry of curcumin: Miniperspective. J. Med. Chem..

[98] Chung S.S., Dutta P., Chard N., Wu Y., Chen Q.-H., Chen G., Vadgama J. (2019). A novel curcumin analog inhibits canonical and non-canonical functions of telomerase through STAT3 and NF-κB inactivation in colorectal cancer cells. Oncotarget.

[99] Den Hartogh D.J., Gabriel A., Tsiani E. (2020). Antidiabetic Properties of Curcumin I: Evidence from In vitro Studies. Nutrients.

[100] Lee S.E., Park H.R., Jeon S., Han D., Park Y.S. (2020). Curcumin attenuates acrolein-induced COX-2 expression and prostaglandin production in human umbilical vein endothelial cells. J. Lipid Atheroscl..

[101] Da Silva A.C., De Freitas Santos P.D., Do Prado Silva J.T., Leimann F.V., Bracht L., Gonçalves O.H. (2018). Impact of curcumin nanoformulation on its antimicrobial activity. Trends Food Sci. Technol..

[102] Huang F., Gao Y., Zhang Y., Cheng T., Ou H., Yang L., Liu J., Shi L., Liu J. (2017). Silver-decorated polymeric micelles combined with curcumin for enhanced antibacterial activity. ACS Appl. Mater. Interfaces.

[103] Zaharieva M.M., Kroumov A.D., Dimitrova L., Tsvetkova I., Trochopoulos A., Konstantinov S.M., Reinhold Berger M., Momchilova M., Yoncheva K., Miladinov Najdenski H. (2019). Micellar curcumin improves the antibacterial activity of the alkylphosphocholines erufosine and miltefosine against pathogenic *Staphyloccocus aureus* strains. Biotechnol. Biotechnol. Equip..

[104] Naseri S., Darroudi M., Aryan E., Gholoobi A., Rahimi H.R., Ketabi K., Movaqar A., Abdoli M., Gouklani H., Teimourpour R. (2017). The Antiviral Effects of Curcumin Nanomicelles on the Attachment and Entry of Hepatitis C Virus. Iran. J. Virol..

[105] Yang Q.Q., Farha A.K., Kim G., Gul K., Gan R.Y., Corke H. (2020). Antimicrobial and anticancer applications and related mechanisms of curcumin-mediated photodynamic treatments. Trends Food Sci. Technol..

[106] Hewlings S., Kalman D. (2017). Curcumin: A review of its’ effects on human health. Foods.

[107] Rafiee Z., Nejatian M., Daeihamed M., Jafari S.M. (2019). Application of different nanocarriers for encapsulation of curcumin. Crit. Rev. Food Sci. Nutr..

[108] Rajasekar A. (2015). Facile synthesis of curcumin nanocrystals and validation of its antioxidant activity against circulatory toxicity in Wistar rats. J. Nanosci. Nanotechnol..

[109] Farhood B., Mortezaee K., Goradel N.H., Khanlarkhani N., Salehi E., Nashtaei M.S., Najafi M., Sahebkar A. (2019). Curcumin as an anti-inflammatory agent: Implications to radiotherapy and chemotherapy. J. Cell. Physiol..

[110] Banez M.J., Geluz M.I., Chandra A., Hamdan T., Biswas O.S., Bryan N.S., Von Schwarz E.R. (2020). A systemic review on the antioxidant and anti-inflammatory effects of resveratrol, curcumin, and dietary nitric oxide supplementation on human cardiovascular health. Nutr. Res..

[111] Nahar P.P., Slitt A.L., Seeram N.P. (2015). Anti-inflammatory effects of novel standardized solid lipid curcumin formulations. J. Med. Food.

[112] Suresh S., Sankar P., Telang A.G., Kesavan M., Sarkar S.N. (2018). Nanocurcumin ameliorates Staphylococcus aureus-induced mastitis in mouse by suppressing NF-κB signaling and inflammation. Int. Immunopharmacol..

[113] Kuttan R., Sudheeran P.C., Josph C.D. (1987). Turmeric, and curcumin as topical agents in cancer therapy. Tumori.

[114] Perera W.P.T.D., Dissanayake R.K., Ranatunga U.I., Hettiarachchi N.M., Perera K.D.C., Unagolla J.M., DeSilva R.T., Pahalagedara L.R. (2020). Curcumin loaded zinc oxide nanoparticles for activity-enhanced antibacterial and anticancer applications. RSC Adv..

[115] Tan B., Norhaizan M.E. (2019). Curcumin combination chemotherapy: The implication and efficacy in cancer. Molecules.

[116] Hotsumi M., Tajiri M., Nikaido Y., Sato T., Makabe K., Konno H. (2019). Design, synthesis, and evaluation of a water soluble C5-monoketone type curcumin analogue as a potent amyloid β aggregation inhibitor. Bioorg. Med. Chem. Lett..

[117] Basniwal R.K., Khosla R., Jain N. (2014). Improving the anticancer activity of curcumin using nanocurcumin dispersion in water. Nutr. Cancer.

[118] Yallapu M.M., Nagesh P.K.B., Jaggi M., Chauhan S.C. (2015). Therapeutic applications of curcumin nanoformulations. AAPS J..

[119] Khan M.N., Haggag Y.A., Lane M.E., Mccarron P.A., Tambuwala M.M. (2018). Polymeric nano-encapsulation of curcumin enhances its anticancer activity in breast (MDA-MB231) and lung (A549) cancer cells through reduction in expression of HIF-1α and nuclear p65 (REL A). Curr. Drug Deliv..

[120] Baghi N., Bakhshinejad B., Keshavarz R., Babashah S., Sadeghizadeh M. (2018). Dendrosomal nanocurcumin and exogenous p53 can act synergistically to elicit anticancer effects on breast cancer cells. Gene.

[121] Arya G., Das M., Sahoo S.K. (2018). Evaluation of curcumin loaded chitosan/PEG blended PLGA nanoparticles for effective treatment of pancreatic cancer. Biomed. Pharmacother..

[122] Paulraj F., Abas F., Lajis N.H., Othman I., Naidu R. (2019). Molecular Pathways Modulated by Curcumin Analogue, Diarylpentanoids in Cancer. Biomolecules.

[123] Fonseca-Santos B., Dos Santos A.M., Rodero C.F., Gremião M.P.D., Chorilli M. (2016). Design, characterization, and biological evaluation of curcumin-loaded surfactant-based systems for topical drug delivery. Int. J. Nanomed..

[124] Biswas A.K., Islam M.R., Choudhury Z.S., Mostafa A., Kadir M.F. (2014). Nanotechnology based approaches in cancer therapeutics. Adv. Natural Sci. Nanosci. Nanotechnol..

[125] Verma K., Tarafdar A., Kumar D., Kumar Y., Rana J.S., Badgujar P.C. (2022). Formulation and characterization of nano-curcumin fortified milk cream powder through microfluidization and spray drying. Food Res. Int..

[126] Atia M.M., Abdel-Tawab H.S., Mostafa A.M., Mobarak S.A. (2022). Nanocurcumin and curcumin prevent N, N′-methylenebisacrylamide-induced liver damage and promotion of hepatic cancer cell growth. Sci. Rep..

[127] Zou P., Zhang J., Xia Y., Kanchana K., Guo G., Chen W. (2015). ROS generation mediates the anti-cancer effects of WZ35 via activating JNK and ER stress apoptotic pathways in gastric cancer. Oncotarget.

[128] Quispe C., Herrera-Bravo J., Khan K., Javed Z., Semwal P., Painuli S., Sharifi-Rad J. (2022). Therapeutic applications of curcumin nanomedicine formulations in cystic fibrosis. Prog. Biomater..

[129] Rajalakshmi N., Dhivya S. (2018). A Review on the preparation methods of Curcumin Nanoparticles. PharmaTutor.

[130] Manikandan S., El Mabrouk K., Ballamurugan A.M. (2022). Synthesis of Nanocurcumin and Evaluation of its Properties for Biomedical Applications. Trends Biomater. Artif. Organs.

[131] Mukerjee A., Vishwanatha J.K. (2009). Formulation, characterization and evaluation of curcumin-loaded PLGA nanospheres for cancer therapy. Anticancer Res..

[132] Mathew A., Fukuda T., Nagaoka Y. (2012). Curcumin loaded-PLGA nanoparticles conjugated with Tet-1 peptide for potential use in Alzheimer’s disease. PLoS ONE..

[133] Yallapu M.M., Jaggi M., Chauhan S.C. (2010). beta-Cyclodextrin-curcumin self-assembly enhances curcumin delivery in prostate cancer cells. Colloids Surf B Biointerfaces.

[134] Yallapu M.M., Ebeling M.C., Chauhan N., Jaggi M., Chauhan S.C. (2011). Interaction of curcumin nanoformulations with human plasma proteins and erythrocytes. Int. J. Nanomed..

[135] He Y., Huang Y., Cheng Y. (2010). Structure Evolution of Curcumin Nano-precipitation from a Micromixer. Cryst. Growth Des..

[136] Hettiarachchi S.S., Dunuweera S.P., Dunuweera A.N., Rajapakse R.G. (2021). Synthesis of curcumin nanoparticles from raw turmeric rhizome. ACS Omega.

[137] Maleki Dizaj S., Alipour M., Dalir Abdolahinia E., Ahmadian E., Eftekhari A., Forouhandeh H., Zununi Vahed S. (2022). Curcumin nanoformulations: Beneficial nanomedicine against cancer. Phytother. Res..

[138] Tabanelli R., Brogi S., Calderone V. (2021). Improving curcumin bioavailability: Current strategies and future perspectives. Pharmaceutics.

[139] Dutta B., Shelar S.B., Rajan V., Checker S., Barick K.C., Pandey B.N., Hassan P.A. (2022). Gelatin grafted Fe_3_O_4_ based curcumin nanoformulation for cancer therapy. J. Drug Deliv. Sci. Technol..

[140] Wang P., Zhang L., Peng H., Li Y., Xiong J., Xu Z. (2013). The formulation and delivery of curcumin with solid lipid nanoparticles for the treatment of on non-small cell lung cancer both in vitro and in vivo. Mater. Sci. Eng..

[141] Abdellah A.M., Sliem M.A., Bakr M., Amin R.M. (2018). Green synthesis and biological activity of silver–curcumin nanoconjugates. Future Med. Chem..

[142] Elbialy N.S., Abdelfatah E.A., Khalil W.A. (2019). Antitumor activity of curcumin-green synthesized gold nanoparticles: *In vitro* study. BioNanoScience.

[143] Chaurasia S., Chaubey P., Patel R.R., Kumar N., Mishra B. (2016). Curcumin-polymeric nanoparticles against colon-26 tumor-bearing mice: Cytotoxicity, pharmacokinetic and anticancer efficacy studies. Drug Dev. Ind. Pharm..

[144] Liu R., Pei Q., Shou T., Zhang W., Hu J., Li W. (2019). Apoptotic effect of green synthesized gold nanoparticles from Curcuma wenyujin extract against human renal cell carcinoma A498 cells. Int. J. Nanomed..

[145] Thadakapally R., Aafreen A., Aukunuru J., Habibuddin M., Jogala S. (2016). Preparation and characterization of PEG-albumin-curcumin nanoparticles intended to treat breast cancer. Indian J. Pharm. Sci..

[146] Al-Ani L.A., Yehye W.A., Kadir F.A., Hashim N.M., AlSaadi M.A., Julkapli N.M., Hsiao V.K. (2019). Hybrid nanocomposite curcumin-capped gold nanoparticle-reduced graphene oxide: Antioxidant potency and selective cancer cytotoxicity. PLoS ONE.

[147] Ayubi M., Karimi M., Abdpour S., Rostamizadeh K., Parsa M., Zamani M., Saedi A. (2019). Magnetic nanoparticles decorated with PEGylated curcumin as dual targeted drug delivery: Synthesis, toxicity, and biocompatibility study. Mater. Sci. Eng. C.

[148] Saikia C., Das M.K., Ramteke A., Maji T.K. (2017). Controlled release of curcumin from thiolated starch-coated iron oxide magnetic nanoparticles: An *in vitro* evaluation. Int. J. Polym. Mater. Polym. Biomat..

[149] Zhou J., Cao Z., Panwar N., Hu R., Wang X., Qu J., Yong K.T. (2017). Functionalized gold nanorods for nanomedicine: Past, present, and future. Coord. Chem. Rev..

[150] Daniels A.N., Singh M. (2019). Sterically stabilized siRNA: Gold nanocomplexes enhance c-MYC silencing in a breast cancer cell model. Nanomedicine.

[151] Mbatha L.S., Maiyo F., Daniels A., Singh M. (2021). Dendrimer-coated Gold Nanoparticles for Efficient Folate-Targeted mRNA Delivery in vitro. Pharmaceutics.

[152] Rejinold N.S., Thomas R.G., Muthiah M., Chennazhi K., Manzoor K., Park I.-K., Jeong Y.Y., Jayakumar R. (2015). Anti-cancer, pharmacokinetics, and tumor localization studies of pH-, RF-and thermo-responsive nanoparticles. Int. J. Biol. Macromol..

[153] Nambiar S., Osei E., Fleck A., Darko J., Mutsaers A.J., Wettig S. (2018). Synthesis of curcumin-functionalized gold nanoparticles and cytotoxicity studies in human prostate cancer cell line. Appl. Nanosci..

[154] Ombredane A.S., Silva V.R., Andrade L.R., Pinheiro W.O., Simonelly M., Oliveira J.V., Joanitti G.A. (2021). *In Vivo* efficacy and toxicity of curcumin nanoparticles in breast cancer treatment: A systematic review. Front. Oncol..

[155] ClinicalTrials.gov. https://www.clinicaltrials.gov/.

